# Evaluation of Serum YKL-40 in Canine Multicentric Lymphoma: Clinical and Diagnostic Implications

**DOI:** 10.3390/ani14233391

**Published:** 2024-11-25

**Authors:** Chien-Chun Kuo, Jih-Jong Lee, Shang-Lin Wang, Yuan-Yuan Xia, Albert Taiching Liao

**Affiliations:** 1 Department of Veterinary Medicine, School of Veterinary Medicine, National Taiwan University, No. 1 Sec. 4, Roosevelt Rd., Taipei City 106319, Taiwan; d08629001@ntu.edu.tw (C.-C.K.); f05629021@ntu.edu.tw (Y.-Y.X.); 2 Institute of Veterinary Clinical Science, School of Veterinary Medicine, National Taiwan University, No. 1, Sec. 4, Roosevelt Rd., Taipei City 106319, Taiwan; jacklee@ntu.edu.tw (J.-J.L.); shanglinwang@ntu.edu.tw (S.-L.W.); 3 National Taiwan University Veterinary Hospital, College of Bioresources and Agriculture, National Taiwan University, No. 153, Sec. 3, Keelung Rd., Taipei City 106328, Taiwan

**Keywords:** canine multicentric lymphoma, biomarker, YKL-40, prognosis

## Abstract

YKL-40, a secretory glycoprotein, is known as a prognostic biomarker in human cancers, but its role in canine multicentric lymphoma is not well understood. This study aimed to investigate serum YKL-40 levels in thirty dogs with multicentric lymphoma to determine their prognostic value, association with patient characteristics, and potential to predict chemotherapy response. Serum samples were collected before, during, and after chemotherapy, and YKL-40 level was measured using ELISA. The results showed that the pretreatment serum YKL-40 levels were significantly higher in dogs with multicentric lymphoma (394.0 pg/mL, *n =* 30) than in healthy controls (218.6 pg/mL, *n =* 11) (*p =* 0.012). While a cutoff value of 445.1 pg/mL was observed, further studies are needed to clarify its diagnostic utility. Dogs with clinical stage V had the highest YKL-40 levels (*p =* 0.027), potentially reflecting disease severity. Furthermore, YKL-40 levels decreased after chemotherapy (*p =* 0.030). However, YKL-40 levels showed no significant association with progression-free survival (PFS) (HR = 0.93, *p =* 0.830) or overall survival (OS) (HR = 0.99, *p =* 0.267). In conclusion, serum YKL-40 levels may potentially detect the disease severity, but its prognostic role remains uncertain. Further studies are required to evaluate serum YKL-40 levels as a potential indicator of treatment response or disease recurrence.

## Simple Summary

Canine multicentric lymphoma is a common neoplasm in dogs, and there is a need to discover diagnostic and prognostic biomarkers to aid veterinarians and pet owners in decision making. Although some protein biomarkers have been identified in the blood of dogs with multicentric lymphoma, there is still a need for new biomarkers are still required to predict the treatment outcome. This study investigated a novel protein biomarker called YKL-40, which is regarded as a prognostic biomarker in human cancers. YKL-40 could be detected in the blood or neoplastic cells. This study assessed the diagnostic and prognostic abilities of blood YKL-40. The findings from thirty dogs with multicentric lymphoma suggest that serum YKL-40 had the disease’s severity in canine multicentric lymphoma. However, its ability to predict clinical outcome remains uncertain. Further large-scale studies are required to clarify the role of YKL-40 in canine multicentric lymphoma.

## 1. Introduction

YKL-40, also known as Chitinase-3-like protein 1 (CH3IL1), is a secretory glycoprotein with chitinase-like characteristics but lacking chitinase activity [1]. It is named YKL-40 because its molecular weight is 40 kDa, and the three N-terminal amino-acid residues are tyrosine (Y), lysine (K), and leucine (L). In humans, YKL-40 has been associated with various cancers as it could be expressed by tumor and inflammatory cells [2], including macrophages [3], neutrophils [4], and mast cells [5]. The biological role of YKL-40 in cancer is still under investigation, but it may be involved in cancer cell proliferation, survival, immune cell activation, and differentiation [6,7]. YKL-40 has been reported as a blood biomarker for various human cancers, including melanoma, glioblastoma, gastrointestinal, ovarian, lung, and urologic cancer [6,7,8]. In human hematologic malignancies, YKL-40 expression has been observed in diffuse large B-cell lymphoma (DLBCL) [9,10] and cutaneous T-cell lymphoma [11,12]. Elevated levels of blood YKL-40 have been correlated with disease severity, such as in the advanced clinical stage of Hodgkin’s lymphoma [13], DLBCL [10], and cutaneous T-cell lymphoma [11,12]. Higher levels of blood YKL-40 are also associated with unfavorable outcomes in non-Hodgkin’s lymphoma [14] and DLBCL [10,15].

Multicentric lymphoma is the most common neoplasm in dogs, primarily involving the lymph nodes [16,17]. Diagnosis typically involves histopathological examination or flow cytometry of fine needle aspiration specimens combined with immunophenotyping [17,18,19]. Common World Health Organization (WHO) subtypes include DLBCL [16,20], peripheral T-cell lymphoma, not otherwise specified (PTCL-NOS), marginal zone lymphoma (MZL), and T-zone lymphoma (TZL). Prognostic factors for canine multicentric lymphoma include histopathological classification [21], immunophenotype [22,23,24], WHO clinical stage [22,24,25,26], substage [22,24,26], chemotherapy regimen [21,27], response to therapy [26], anemia [24,27,28], and neutrophilia [27].

Various blood proteins have been explored as potential biomarkers for canine multicentric lymphoma. Proteins such as TK1 [29,30,31,32,33], CRP [30,31,34,35,36], MCP-1 [37], IL-6 [37], IL-10 [37], and haptoglobin [38,39] could be elevated in dogs with multicentric lymphomas, but their prognostic value is still under investigation. Different immunophenotypes may have different biomarkers. For example, B-cell lymphoma may exhibit higher levels of IL-10 [37] and TK1 [33,40] than T-cell lymphoma, while T-cell lymphoma may show higher levels of IL-6 [37]. Elevated blood protein levels may be correlated with advanced clinical stages, such as MCP-1 [41], CRP [36], and VEGF [42]. Elevated blood protein levels may also be linked to poor prognosis, such as increased MCP-1 levels in dogs with multicentric lymphoma following CHOP chemotherapy being associated with shorter disease-free intervals [41]. Although various blood proteins may be elevated in dogs with multicentric lymphoma, their prognostic value remains inconclusive. For instance, TK1 levels do not correlate with the prognosis of DLBCL following CHOP-based chemotherapy [43] and the survival of multicentric lymphoma [40]. CRP concentration cannot be used to monitor disease status [34]. Therefore, further investigation into new blood biomarkers is needed to predict prognosis and monitor the disease progression of canine multicentric lymphoma.

Previous studies have indicated that dogs with various cancers, such as lymphoma, mast cell tumors, and mammary gland tumors, exhibit elevated levels of blood YKL-40. These elevated levels of blood YKL-40 are associated with a higher chance of disease recurrence [44]. However, the role of YKL-40 in predicting the clinical outcome of canine multicentric lymphoma has not been investigated. Therefore, this study aimed to investigate the level of serum YKL-40 in dogs with multicentric lymphoma and its correlation with disease characteristics, including patient signalment, immunophenotype, clinical stage, disease progression, and survival time, to determine its prognostic value. This study also aimed to assess its potential in predicting the response to chemotherapy.

## 2. Materials and Methods

### 2.1. Patient

Dogs diagnosed with multicentric lymphoma who were to be treated with chemotherapy were enrolled in this study conducted from August 2019 to July 2023 at the National Taiwan Veterinary Hospital (NTUVH). The inclusion criteria included a cytologic and histologic diagnosis of multicentric lymphoma through an affected lymph node, along with immunophenotyping to determine the immunophenotypes. The exclusion criteria were inconclusive immunophenotyping results, small cell lymphoma, indolent lymphoma, and dogs with prior prednisolone or chemotherapy treatment. The clinical stage and substage were evaluated and determined by the clinician based on the World Health Organization (WHO) clinical stage [25] and substage [45]. Age, gender, neutered status, weight, breed, diagnostic method, immunophenotype, complete blood count (CBC), serum biochemistry panel, and time of diagnosis were recorded for each patient. The number of dogs was included was based on availability during the study period. Thirty dogs diagnosed with multicentric lymphoma were enrolled.

Eleven healthy dogs were included in the control group. These dogs underwent health exams at NTUVH during the study period. These dogs were confirmed to be healthy based on thoracic radiography, abdominal ultrasound, and hematological examinations. The serum sampling procedures were consistent with those used for the dogs with lymphoma in the study.

The experimental protocol was ethically reviewed and approved by the Institutional Animal Care and Use Committee of the National Taiwan University (IACUC approval number: NTU-109-EL-00169). Informed consent forms were obtained from the owners of all enrolled dogs.

### 2.2. Treatment, Outcome, and Data Collection

Twenty-eight dogs received at least 15 weeks of CHOP-based chemotherapy. The 15-week CHOP-based chemotherapy protocol consisted of vincristine at 0.7 mg/m^2^ in the first week, cyclophosphamide at 250 mg/m^2^ in the second week, and doxorubicin at 30 mg/m^2^ in the third week, repeated for four cycles. Prednisolone was prescribed at 1 to 2 mg/kg daily. L-asparaginase could be substituted for cyclophosphamide, as decided by the clinician. In the case of chemotherapy resistance, rescue chemotherapy may be administered, including lomustine, cytarabine, and L-asparaginase. Mitoxantrone could be substituted for doxorubicin if there were concerns about cardiac disease. The remaining two dogs were excluded as they did not undergo treatment.

Response to chemotherapy was assessed using the response evaluation criteria for peripheral nodal lymphoma in dogs (V1.0) of the Veterinary Comparative Oncology Group (VCOG) [46]. Complete remission (CR) was defined as all lymph nodes returning to a non-pathologic size. Partial remission (PR) was defined as at least a 30% decrease in the mean of the longest diameters of up to five affected lymph nodes, compared with the baseline mean sum of the longest diameters. Progressive disease (PD) was defined as the appearance of one or more new lesions or at least a 20% increase in the sum of the longest diameters of up to five affected lymph nodes. Stable disease (SD) was defined as a measurable tumor burden not meeting the criteria for PR or PD. After completing the chemotherapy protocol, dogs were asked to be brought for revisits every three months to evaluate the size of peripheral lymph nodes. If lymphadenopathy was observed, cytological examination was performed to confirm the recurrence of lymphoma. The recurrence time was documented if recurrence was confirmed and chemotherapy was reinduced. Dogs were monitored until death or loss to follow-up.

Data were extracted from the patients’ medical records. Variables collected included age, sex, breed, body weight, immunophenotype, WHO clinical stage, substage, presence of leukocytosis, and presence of anemia. Leukocytosis was defined as a white blood cell count exceeding 17,000 cells per microliter, and anemia was defined as a hematocrit level below 37%. Clinical outcomes documented included time of disease progression, time of relapse, and time of death. Progression-free survival (PFS) was defined as the interval from initiation of chemotherapy to the first occurrence of disease progression, relapse, or death due to any cause, whichever came first. Dogs were censored in the analysis if the disease progression or death event was not identified. Overall survival (OS) was defined as the time from diagnosis to death from any cause, with censoring applied to dogs alive or loss to follow-up until the end of the study. For instances lacking documentation of death or relapse event on in the medical record, follow-up communication was conducted via phone call with the owners to gather relevant information.

### 2.3. Canine YKL-40 Immunoassays

Serum samples from patients were collected at different timepoints, including at pretreatment, during treatment, and post-treatment follow-up visits, and upon recurrence. Only representative time points associated with tumor change were selected for the analysis of serum YKL-40 levels; these included pretreatment, one week post-treatment, one week before disease progression, at the time of progressive disease, during post-treatment follow-up, and at the time of disease relapse. The serum samples from the health control group were collected during their health examination. Blood was drawn into a serum separator tube (Becton Dickinson, Franklin Lakes, NJ, USA) and allowed to coagulate before being centrifugation at 4000 rpm for 10 min at 4 °C. The supernatant was transferred into a microcentrifuge tube. The serum samples were stored at −80 °C until analysis.

A commercial canine sandwich enzyme-linked immunosorbent assay (ELISA) kit (Cat. SL0006Ca, Sunlong Biotech, Hangzhou, China) was used to evaluate the YKL-40 level according to the instruction manual. The assay began with a five-fold dilution of the serum using the sample dilution buffer. Diluted serum samples were loaded into the antibody-precoat microplate and incubated at 37 °C for 30 min. The HRP-conjugate reagent was loaded into the microplate after the wash step with the buffer and then incubated at 37 °C for 30 min. Subsequently, the chromogen solution was loaded into the microplate and incubated at 37 °C for 15 min. The reaction was halted by adding the stop solution, and the absorbance was measured at an optical density of 450 nm using the microplate spectrophotometer (Multiskan GO, Thermos Fisher Scientific Inc., Waltham, MA, USA). All samples were performed in duplicate. The standard curve and the concentration of YKL-40 were computed using SkanIt Software for Microplate Readers (version 7, Thermos Fisher Scientific Inc., Waltham, MA, USA).

### 2.4. Statistical Analysis

Continuous variables conforming to a normal distribution were denoted as mean ± standard deviation. Those deviating from a normal distribution were denoted as median and interquartile range (IQR). Categorical variables were denoted as numbers and frequency. Receiver Operating Characteristic (ROC) curve analysis was used for the differential analysis of YKL-40 concentration between dogs with lymphoma and healthy controls. The Mann–Whitney and Kruskal–Wallis tests were employed to compare continuous variables without normal distribution. Pearson correlation analysis was performed to evaluate the relationship between pretreatment YKL-40 level and continuous patient variable. The Kaplan–Meier method was applied to estimate the PFS and OS functions within the study population and between subgroups, with survival comparison conducted using the log-rank test. Non-parametric tests were conducted to compare serum YKL-40 levels among patients at different time points. The Wilcoxon signed-rank test was employed for paired comparison when corresponding data were available, while the Mann-Whitney U test was utilized for unpaired comparison when most corresponding data were absent. Univariate and multivariate COX proportional hazard regression was applied to analyze the association between subgroup categorization and survival outcomes.

Statistical significance was set at a *p*-value ≤ 0.05. All the statistical analyses were conducted using SPSS Statistics for Macintosh (version 27, IBM Corp., Armonk, NY, USA). The graphs were generated using GraphPad Prism for Macintosh (version 10, GraphPad Software, Boston, MA, USA).

## 3. Results

### 3.1. Patients

Thirty dogs with multicentric lymphoma were enrolled. Pretreatment and subsequent serum samples were collected to analyze the correlation between dog characteristics and serum YKL-40 levels.

The characteristics of these 30 dogs are shown in Table 1 and detailed in Table S1. The median age was 10.5 years, ranging from 3 to 15 years. The were 18 males (2 intact and 16 castrated) and 12 females (1 intact and 11 spayed). The median body weight was 14 kg, ranging from 1.6 to 37.1 kg. These dogs included six mixed-breed dogs, eight Welsh Corgis, two Chihuahuas, and two Miniature Poodles. The remaining 12 dogs were a Beagle, a Chow Chow, a Dachshund, an English Bulldog, a French Bulldog, a Golden Retriever, a Husky, Maltese, an Old English Sheepdog, a Pomeranian, a Shetland Sheepdog, and a Yorkshire Terrier. Six dogs were diagnosed via pathological exam, including five with DLBCL and one with PTCL-NOS. Twenty-four dogs were diagnosed by cytological exam as a median to large lymphoma followed by immunotyping through flow cytometry, including identified twenty B-cells and four T-cells. The predominant immunophenotype was B-cell, accounting for 83% (25/30) of cases, while T-cell lymphoma was identified in 17% (5/30) cases. WHO clinical staging showed one dog in Stage II, six in Stage III, twenty in Stage IV, and three in Stage V. Eighteen dogs were classified as substage a, and 12 as substage b. Leukocytosis was present in 30% (9/30) of dogs, and anemia was present in 50% (15/30).

The control group consisted of 11 healthy dogs, including seven males and four females. The breeds were Labrador Retriever (*n =* 6), Doberman (*n =* 2), German Shepherd (*n =* 1), Chihuahua (*n =* 1), and mixed breed (*n =* 1). The median age was 3 years, ranging from 2 to 12 years. The median body weight was 25.9 kg, ranging from 3.6 to 37.6 kg.

### 3.2. Survival Analysis and Treatment Outcomes in Dogs with Multicentric Lymphoma

In the survival analysis, 30 treatment-naïve dogs were initially considered, but two dogs were excluded as they did not undergo treatment. Therefore, twenty-eight dogs who received at least 15 weeks of CHOP-based chemotherapy were included in the survival analysis. Of these, twenty dogs received CHOP chemotherapy (CHOP group), while eight received CHOP-based chemotherapy with cyclophosphamide substituted with L-asparaginase (LHOP group). There was no significant difference in PFS between the CHOP (PFS of 218 days) and LHOP (PFS of 269 days) groups (*p =* 0.954). Similarly, there was no significant difference in OS between the CHOP (OS of 351 days) and LHOP (OS of 434 days) groups (*p =* 0.678). After receiving CHOP-based chemotherapy, five dogs received rescue chemotherapy, including melphalan, mitoxantrone, lomustine, and chlorambucil.

The median treatment duration for the 28 dogs was 140 days (range: 49 to 424 days). The median PFS was 245 days (range: 49 to 1398 days). The median OS was 351 days (range: 49 to 1398 days). The median follow-up time was 351 days (range: 49 to 1398 days). Three dogs did not experience disease recurrence and were censored from the PFS analysis. Seven dogs were still alive at the end of the study and were censored from the OS analysis.

The PFS and OS for the different subgroups are presented in Table 2. Of the 28 dogs, 73% (22/28) completed the chemotherapy regimen, with a median PFS of 269 days (range: 155 to 1398 days) and a median OS of 533 days (range: 155 to 1398 days). Conversely, 27% (6/28) died because of poor response to chemotherapy, with a median OS of 143 days (range: 49 to 424 days). Among the dogs who completed chemotherapy, 73% (16/22) experienced tumor recurrence, with a median survival time of 349 days (range: 155 to 1142 days), while 27% (6/22) remained recurrence-free, with a median survival time of 918 days (range: 434 to 1398 days). There were significant differences in both PFS (*p =* 0.011) and OS (*p* < 0.001) between patients who completed chemotherapy and those who did not.

The median PFS by clinical stages was 424 days for the combination of stage II + III (*n =* 6), 237 days for stage IV (*n =* 19), and 209 days for stage V (*n =* 3), showing a significant difference (*p =* 0.027). The median PFS differed significantly between substage a (*n =* 18, 279 days) and substage b (*n =* 10, 209 days) (*p =* 0.042). Dogs weighing under 15 kg had a median PFS of 279 days (*n =* 17), while those over 15 kg had a median PFS of 213 days (*n =* 11), showing significant differences (*p =* 0.013). There were significant differences in PFS and OS between dogs with leukocytosis (PFS: 195 days, OS: 241 days) and without leukocytosis (PFS: 269 days, OS: 434 days) (*p =* 0.043 for PFS and *p =* 0.029 for OS). The treatment, outcome, PFS, and OS of individuals are provided in Table S2.

### 3.3. Elevated Serum YKL-40 in Dogs with Multicentric Lymphoma Is Not Correlated with Survival

**Figure 1:**
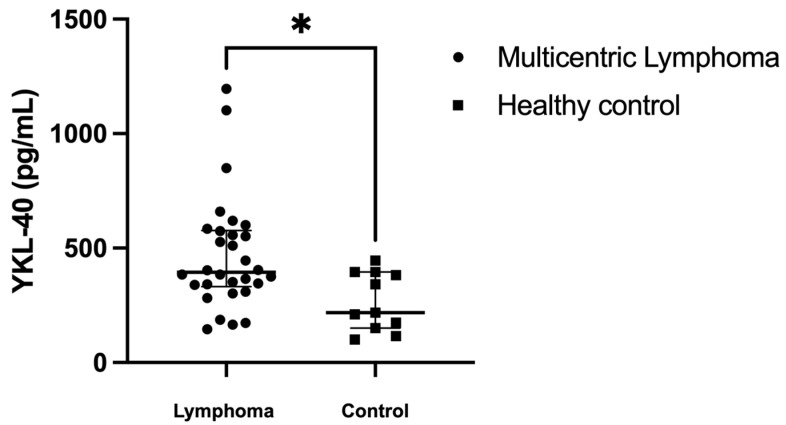
Comparison of serum YKL-40 levels between dogs with multicentric lymphoma and healthy controls. The scatter plot presents individual data points for each group, with the median represented by a central horizontal line and IQR shown around the median. The serum YKL-40 levels significantly differed between the dogs with multicentric lymphoma (*n =* 30) and the healthy control (*n =* 11). * *p =* 0.012.

**Figure 2:**
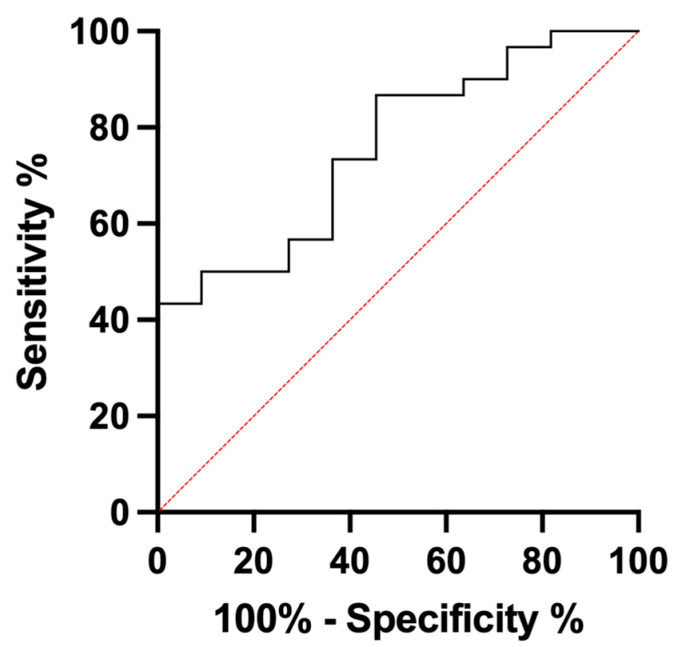
ROC curve of serum YKL-40 levels for differentiating dogs with multicentric lymphoma from healthy dogs. The ROC curve demonstrates the diagnostic accuracy of serum YKL-40 levels in distinguishing between dogs with multicentric lymphoma (*n =* 30) and healthy dogs (*n =* 11). The area under the curve (AUC) is 0.766. A cut-off value for YKL-40 was determined to be 445.1 pg/mL, yielding a sensitivity of 43.3% and a specificity of 100%.

In the analysis of serum YKL-40 levels, dogs with multicentric lymphoma had a median YKL-40 level of 394.0 pg/mL (range: 146 to 1196 pg/mL), which was significantly higher than that of healthy dogs, whose median YKL-40 level was 218.6 pg/mL (range: 101.1 to 445.0 pg/mL) (*p =* 0.012) (Figure 1). The ROC curve analysis showed an area under the curve (AUC) of 0.766, indicating a significant difference between the two groups (*p =* 0.008, 95% CI 0.616–0.917) (Figure 2). The cutoff value of YKL-40 was determined to be 445.1 pg/mL, with a sensitivity of 43.3% and specificity of 100%. Using this cut-off value, 43% (*n =* 13) of dogs with lymphoma exhibited elevated YKL-40 levels, while the remaining 56.7% (*n =* 17) did not.

Pearson correlation analysis showed no significant association between pretreatment YKL-40 levels and continuous patient variables such as age (r = −0.253, *p =* 0.194), body weight (r = 0.075, *p =* 0.704), white blood cell count (WBC) (r = 0.026, *p =* 0.895), neutrophil count (r = −0.199, *p =* 0.311), lymphocyte count (r = 0.092, *p =* 0.641), monocyte count (r = −0.012, *p =* 0.952), or packed cell volume (PCV) (r = −0.183, *p =* 0.351).

**Figure 3:**
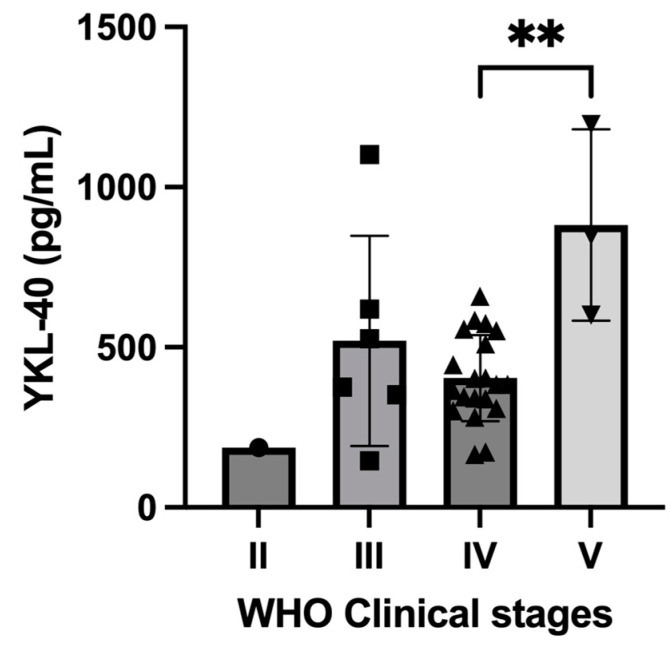
Scatter plot with a bar chart showing pretreatment serum YKL-40 levels in dogs with different WHO clinical stages. Each data point represents an individual dog, with dots, squares, triangles, and inverted triangles representing dogs at clinical stages II, III, IV, and V, respectively. The median values for each stage are shown at the top of the bars, and IQR is represented as horizontal lines around the median. Significant differences are found between the dogs with stage IV and stage V. ** *p =* 0.035.

The serum YKL-40 levels among different subgroups of 30 treatment-naïve dogs are shown in Table 3. Dogs in clinical stage V had the highest median of YKL-40 level. A significant difference in serum YKL-40 was found between dogs in clinical stage V (*n =* 3, median YKL-40 = 849.1 pg/mL) and clinical stage IV (*n =* 20, median YKL-40 = 384.9 pg/mL) dogs (*p =* 0.035), but not between stage V and stage III (*n =* 6, median YKL-40 = 451.4 pg/mL) dogs (*p =* 0.463) (Figure 3). No significant difference was observed in YKL-40 levels between WHO substage a (*n =* 18, median YKL-40 = 403.5 pg/mL) and substage b (*n =* 12, median YKL-40 = 380.3 pg/mL) dogs (*p =* 0.787). YKL-40 levels showed no significant difference when comparing younger (under ten years old, *n =* 12, median YKL-40 = 415.1 pg/mL) with older dogs (over ten years, *n =* 18, median YKL-40 = 377.2 pg/mL) (*p =* 0.465), male dogs (*n =* 18, median YKL-40 = 424.6 pg/mL) with female dogs (*n =* 12, median YKL-40 = 343.9 pg/mL) (*p =* 0.172), non-neutered (*n =* 3, median YKL-40 = 309.8 pg/mL) with neutered dogs (*n =* 27, median YKL-40 = 403.1 pg/mL) (*p =* 0.744), purebred dogs (*n =* 24, median YKL-40 = 384.9 pg/mL) with mixed-breed dogs (*n =* 6, median YKL-40 = 480.5 pg/mL) (*p =* 0.432), or dogs weighing under 15 kg (*n =* 17, median YKL-40 = 403.1 pg/mL) with dogs weighing over 15 kg (*n =* 13, median YKL-40 = 384.9 pg/mL) (*p =* 0.837). Moreover, YKL-40 levels were not significantly different between B-cell (*n =* 25, median YKL-40 = 384.9 pg/mL) and T-cell lymphomas (*n =* 5, median YKL-40 = 527.0 pg/mL) (*p =* 0.448).

In the survival analysis of the 28 dogs with multicentric lymphoma, YKL-40 levels were not significantly correlated with PFS (r = −0.3, *p =* 0.121) or OS (r = −0.201, *p =* 0.305). Dogs with YKL-40 levels below the cutoff of 445.1 pg/mL (*n =* 15, median PFS = 237 days) showed no significant difference in PFS compared with those with levels above the cut-off (*n =* 13, median PFS = 269 days) (*p =* 0.810). Similarly, there was no significant difference in OS between dogs with YKL-40 levels below 445.1 pg/mL (*n =* 15, median OS = 350 days) and those with levels above 445.1 pg/mL (*n =* 13, median OS = 533 days) (*p =* 0.280) (Table 2). The serum YKL-40, characteristics, and outcome of individuals are provided in Tables S1–S4.

### 3.4. In the Longitudinal Study, Serum YKL-40 Showed Reduction After Chemotherapy but Did Not Clearly Predict Treatment Outcomes in Multicentric Lymphoma

**Figure 4:**
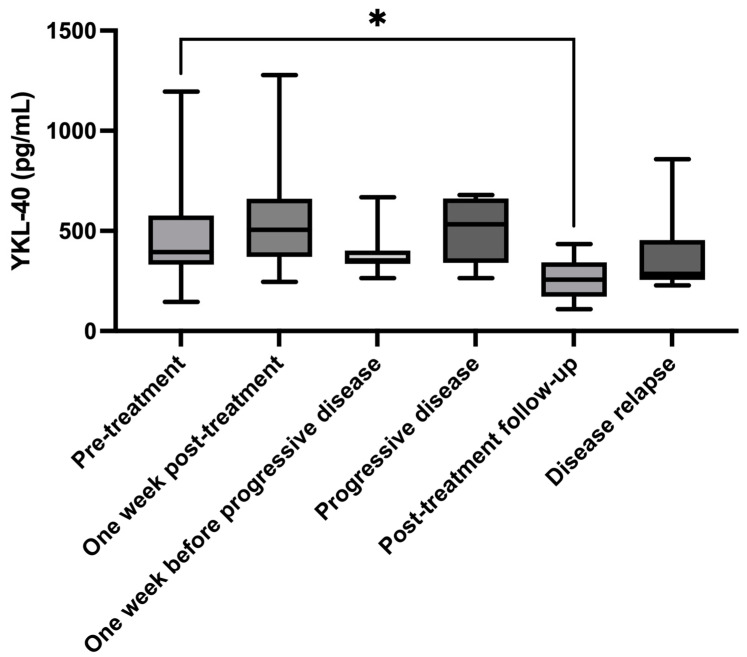
Box and whiskers plot showing comparisons of serum YKL-40 levels across different treatment time points. The plot presents the distribution of serum YKL-40 levels at each time point, with boxes indicating the IQR around the median, and whiskers extending to minimum and maximum values. Significant differences were observed between pretreatment and post-treatment follow-up. * *p =* 0.0312.

Representative serum samples collected at different time points corresponding to disease progression or remission were analyzed to observe YKL-40 fluctuations. These time points included pretreatment, one week post-treatment, one week before disease progression, progressive disease, post-treatment follow-up, and at the time of disease relapse. The number of cases and serum YKL-40 analysis at different time points are detailed in Table 4. The illustration and comparisons of serum YKL-40 levels across different treatment time points are shown in Figure 4.

A paired analysis of serum YKL-40 levels between pretreatment (*n =* 16, median YKL-40 = 477.8 pg/mL, IQR: 359.7–590.0 pg/mL) and one week post-treatment (*n =* 16, median YKL-40 = 506.2 pg/mL, IQR: 370.7–660.9 pg/mL) was not statistically significant (*p =* 0.4037). Serum YKL-40 levels at the time of progressive disease (*n =* 7, median YKL-40 = 551.2, IQR: 317.3–664.2 pg/mL) were higher compared with levels one week before disease progression (*n =* 7, median YKL-40 = 353.4, IQR: 335.4–401.7 pg/mL), but this increase was not significant (*p =* 0.3750). A significant reduction in serum YKL-40 was observed during post-treatment follow-up (*n =* 6, median YKL-40 = 256.8 pg/mL, IQR: 171.8–342.7 pg/mL) compared with pretreatment levels (*n =* 6, median YKL-40 = 501.2 pg/mL, IQR: 390.2-912.3 pg/mL) (*p =* 0.0312). Serum YKL-40 levels at disease relapse (*n =* 6, median YKL-40 = 285.4 pg/mL, IQR: 256.0-453.8 pg/mL) were lower than pretreatment levels (*n =* 6, median YKL-40 = 477.8 pg/mL, IQR: 380.2–642.8 pg/mL) but without statistical significance (*p =* 0.0625). An unpaired analysis between post-treatment follow-up (*n =* 6, median YKL-40 = 256.8 pg/mL, IQR: 171.8–342.7 pg/mL) and disease relapse (*n =* 6, median YKL-40 = 285.4 pg/mL, IQR: 256.0-453.8 pg/mL) showed no significant difference (*p =* 0.3939). The serum YKL-40 at representative time points of individuals is provided in Table S3.

### 3.5. Univariate Analysis Revealed Potential Prognostic Factors in Canine Lymphoma, But Multivariate Analysis Showed Limited Predictive Significance

In the univariate analysis of 28 dogs with follow-up, body weight (HR [95% CI] = 1.082 [1.02–1.147], *p =* 0.009), WHO clinical stage (II/III vs. V; HR [95% CI] = 7.907 [1.566–39.929], *p =* 0.012), substage (a vs. b; HR [95% CI] = = 2.310 [1.008–5.3], *p =* 0.048), and leukocytosis (leukocytosis vs. normal, HR [95% CI] = 2.386 [1.003–5.675], *p =* 0.049) were significantly associated with PFS. Leukocytosis (leukocytosis vs. normal, HR [95% CI] = 2.845 [1.071–7.56], *p =* 0.036) was significantly associated with OS. Dogs with body weights below 15 kg had a median PFS of 279 days, significantly different from that of dogs over 15 kg, who had a median PFS of 213 days (HR [95% CI] = 2.92 [1.213–7.333], *p =* 0.017). Dogs at WHO clinical stage V had a median PFS of 209 days, whereas those at WHO clinical stage II/III had a median PFS of 424 days (HR [95% CI] = 7.907 [1.566–39.929], *p =* 0.012). Dogs at substage a had a median PFS of 279 days, whereas those at substage b had a median PFS of 209 days (HR [95% CI] = 2.31 [1.008–5.3], *p =* 0.048). Dogs with leukocytosis had a median PFS of 195 days, while those without leukocytosis had a median PFS of 269 days (HR [95% CI] = 2.386 [1.003–5.675], *p =* 0.049). However, in multivariate analysis, these factors remained insignificant for predicting PFS.

## 4. Discussion

This study investigated the correlation between YKL-40 serum concentrations and disease status in canine multicentric lymphoma undergoing CHOP-based chemotherapy. The aims were to evaluate whether YKL-40 has prognostic value and is associated with patient characteristics, as well as to assess whether YKL-40 can predict the response to CHOP chemotherapy. The study population consisted of dogs with medium to large cell multicentric lymphomas. The findings indicated that dogs with multicentric lymphoma had elevated serum YKL-40 levels compared with healthy controls. Notably, dogs at clinical stage V exhibited higher YKL-40 concentrations than those at other stages. However, our analysis revealed no correlation between YKL-40 level and various clinical variables, including signalment, substage, immunophenotype, progression-free survival (PFS), or overall survival (OS), suggesting a complex relationship between YKL-40 expression and the pathogenesis of multicentric lymphoma.

The prognosis of canine multicentric lymphoma could be associated with a spectrum of factors, including histopathological classification [21], immunophenotype [22,23,24], WHO clinical stage [22,24,25,26], substage [22,24,26,45], chemotherapy regimen [21,27], response to therapy [26], and presence of anemia [24,27,28] and neutrophilia [27]. Our univariate analysis highlighted body weight, clinical stage, and substage as factors associated with PFS, while leukocytosis was linked with OS. Consistently with previous studies, certain prognostic factors such as WHO clinical stage [22,24,25,26] and substage [22,24,26] were significantly correlated with PFS, while leukocytosis was the only factor significantly associated with OS. The response to treatment in our study was significantly correlated with PFS and OS, consistently with a prior study [26]. Furthermore, our analysis suggested that elevated white blood cell counts, including neutrophils and monocytes, were the indicators of shorter disease-free survival.

The study population consisted of dogs diagnosed with medium to large cell multicentric lymphoma, which indicates high-grade lymphoma. For dogs with pathological diagnoses, DLCBL was the primary pathological classification. In our study, the median survival time was 351 days, which is similar to previous studies in which median survival times were reported as 246 days [26] and 254 days [47]. Specifically, the survival time for our B-cell lymphoma cases was 424 days, which is comparable to the reported survival time for DLBCL, ranging between 292 days [43] and 322 days [27]. Interestingly, the median survival time for our T-cell lymphoma cases was 350 days, exceeding the previously reported 136 days for non-indolent T-cell lymphoma [48]. We suggest that the differences in survival time may be caused by different treatment modalities, leading to differences in outcomes between subgroups. In addition, the body weight of our dog population was lower than in the previous study, and they may received a relatively higher dosage of chemotherapy according to body weight and body surface area. This highlights the potential variability in multicentric lymphoma when evaluating prognosis and treatment outcomes. However, individual variations may be one of the causes of varying treatment responses and outcomes.

Serum YKL-40 levels were elevated in dogs with multicentric lymphoma compared with healthy dogs, consistent with previous research [44]. However, YKL-40 may not be reliable as a diagnostic biomarker for canine multicentric lymphoma because of the low sensitivity of 43.3%, despite a high specificity of 100% at a 445.1 pg/mL cut-off value. The ROC curve analysis showed that YKL-40 has limited sensitivity as a diagnostic biomarker for multicentric lymphoma, which limits its clinical applicability. Additionally, the serum YKL-40 values in our study were higher than those in previous studies [44], possibly due to the use of serum samples rather than plasma samples, which could influence the YKL-40 concentration.

Serum YKL-40 levels were higher in canine multicentric lymphoma with advanced clinical stages, suggesting its potential association with disease progression. This finding is similar to human lymphomas, such as Hodgkin’s lymphoma [13], DLBCL [10], and cutaneous T-cell lymphoma [11,12]. Nonetheless, the limited number of dogs with advanced clinical staging could introduce biases by potentially conflating clinical stage IV and V dogs and thus impacting the interpretation of the data. Therefore, studies with a larger sample size should be conducted to confirm the diagnostic ability of YKL-40.

Our study did not find a significant association between pretreatment serum YKL-40 levels and PFS or OS in canine multicentric lymphoma. This lack of correlation may be due to the heterogeneity of lymphoma or differences in YKL-40 secretion among lymphoma cells, which could affect its role as a prognostic biomarker. The results are similar to those for human Hodgkin’s lymphoma, in which elevated YKL-40 levels are not associated with survival [13]. However, in human non-Hodgkin’s lymphoma, high YKL-40 levels correlate with poor outcomes [10,14,15]. Furthermore, human DLBCL patients who died of the disease had higher levels of YKL-40 in their blood compared to survivors [10]. Because YKL-40 expression in human lymphoma cells is lower than in solid tumors [2], variable YKL-40 secretion by lymphoma cells may contribute to differences in circulating YKL-40 and its prognostic value. This difference may highlight the varied prognostic roles of YKL-40 in different subtypes of lymphoma and patient populations.

The prognosis of multicentric lymphoma is primarily determined by the treatment response, which limits the use of serum proteins as prognostic indicators. Chemotherapy regimens and the treatment response are more critical prognostic indicators [21,26,27]. Despite the elevation of various blood biomarkers in multicentric lymphoma, particularly at advanced clinical stages, their correlation with the prognosis often remains inconclusive. For instance, TK1 levels do not consistently correlate with survival or advanced disease stages despite the increase in multicentric lymphoma [40]. Similarly, in patients with DLBCL undergoing CHOP-based chemotherapy, TK1 activity does not correlate with PFS or achieving a complete response [43]. However, concurrent assessment of multiple biomarkers, such as TK1 and CRP [31], or haptoglobin and CRP [39], has shown greater prognostic potential than individual markers. To enhance the diagnostic and prognostic value of YKL-40, future studies could consider analyzing YKL-40 along with other biomarkers, such as TK1, CRP, and IL-6. Combined biomarker analysis might improve the overall efficacy and reliability of these biomarkers [49,50].

The study also aimed to preliminarily evaluate whether serum YKL-40 levels could indicate disease progression in canine multicentric lymphoma. It was hypothesized that the YKL-40 levels would decrease and align with disease remission. Our findings revealed no significant change in YKL-40 levels one week after the start of treatment, whether complete or partial remission was achieved. A longitudinal assessment of YKL-40 levels before and at the time of progressive disease was conducted to explore its potential as an early biomarker for disease relapse. However, the change in YKL-40 levels was not significant, questioning its value as a therapeutic biomarker compared with measuring tumor size. Nevertheless, the YKL-40 levels significantly decreased during the remission phase after chemotherapy. This decrease may indicate that YKL-40 could reflect treatment success in achieving remission. However, its value as a biomarker for predicting remission or relapse needs further investigation. The absence of increased YKL-40 at relapse compared with pretreatment YKL-40 could indicate that pet owners were detecting disease relapse early, potentially preventing notable disease progression and the subsequent rise in YKL-40 levels. Variability in YKL-40 expression among lymphoma cases might further explain its reduced sensitivity [2]. Interestingly, changes in YKL-40 were noted in T-cell lymphoma with disease changes, indicating its potential specificity in this subtype. Because of the small sample size of T-cell lymphoma cases, a further longitudinal study with a larger sample size is required to validate the serum YKL-40 as a biomarker for treatment response.

The limitations of this study included a relatively small sample size and variability in following treatment protocols. The use of flow cytometry instead of histopathology for diagnosis may have limited our detailed investigation of YKL-40 in multicentric lymphoma. The limited number of cases may affect the statistical power of the findings. Relying on a single biomarker without evaluating other potential biomarkers may have limited the comprehensiveness of our findings. These limitations indicate the need for a more controlled, prospective study with a larger sample size and standardized diagnostic and overall treatment protocols to determine the role of YKL-40 in prognosis and response to therapy.

## 5. Conclusions

In conclusion, dogs with multicentric lymphoma showed elevated serum YKL-40 levels, particularly in advanced clinical stages. The serum YKL-40 levels significantly decreased after the completion of the chemotherapy regimen. However, changes in serum YKL-40 levels during therapy did not correspond to treatment response. The prognostic value of YKL-40 in canine multicentric lymphoma remains uncertain as no significant correlations were found between YKL-40 levels and clinical outcomes or prognostic factors, such as PFS, OS, substage, and immunophenotype. Further studies with larger sample sizes are necessary to clarify the potential role of YKL-40 in disease diagnosis and prognosis.

## Figures and Tables

**Table 1:** The characteristics of the dogs with multicentric lymphoma enrolled in this study (*n =* 30).

Characteristics	Classification	Cases	Median	
Age, years (IQR)		30	10.5 (8.3–12)	
Sex	Male	18		
	Female	12		
Body weight, kg (IQR)		30	14 (1.6–37.1)	
Breed	Mixed	6		
	Corgi	8		
	Chihuahua	2		
	Miniature Poodle	2		
	Others	12		
Pathological diagnosis	DLBCL	5		
	PTCL-NOS	1		
Cytological diagnosis ^1^	B-cell lymphoma	20		
	T-cell lymphoma	4		
Phenotype	B-cell	25		
	T-cell	5		
Clinical stage	II	1		
	III	6		
	IV	20		
	V	3		
Substage	a	18		
	b	12		
Leukocytosis		9		
Anemia		15		

^1^ Median to large cell lymphoma diagnosed via flow cytometry.

**Table 2:** Progression-free survival (PFS) and overall survival (OS) in the subgroups of dogs receiving chemotherapy (*n =* 28).

Characteristics	Classification	Cases	PFS, Median (IQR)	*p* Value	OS, Median (IQR)	*p* Value
Overall		28	245 (195–432)		351 (216–554)	
Body weight	BW < 15 kg	17	279 (211–554)	0.013	533 (267–1120)	0.351
	BW ≥ 15 kg	11	213 (185–287)		350 (224–413)	
Clinical stage	II + III	6	424 (279–434)	0.027	434 (424–645)	0.254
	IV	19	237 (185–356)		350 (224–560)	
	V	3	209 (49–218)		413 (49–413)	
Substage	a	18	279 (213–533)	0.042	434 (234–1120)	0.319
	b	10	209 (195–237)		347 (241–413)	
Immunophenotype	B-cell	23	269 (209–434)	0.785	424 (234–645)	0.846
	T-cell	5	196 (195–218)		350 (347–413)	
Serum YKL-40	<445.1 pg/mL	15	237 (169–434)	0.810	350 (234–434)	0.280
	≥445.1 pg/mL	13	269 (195–356)		533 (347–1120)	
Leukocytosis	No	20	269 (209–434)	0.43	434 (309–1120)	0.29
	Yes	8	195 (93–237)		241 (93–347)	
Anemia	No	15	237 (196–424)	0.816	350 (241–534)	0.772
	Yes	13	287 (185–533)		413 (224–1120)	
Complete treatment	No	6	143 (93–234)	0.011	143 (93–234)	<0.001
	Yes	22	269 (211–533)		533 (347–1120)	

**Table 3:** The serum YKL-40 levels in different characteristic subgroups of the dogs with multicentric lymphoma before treatment (*n =* 30).

Characteristics	Classification	Cases	YKL-40 (pg/mL) Median (IQR)	*p* Value
Age	Age < 10 years	12	415.1 (368.6–582.2)	0.465
	Age ≥ 10 years	18	377.2 (296.5–568.1)	
Sex	Male	18	424.6 (362.5–603.5)	0.172
	Female	12	343.9 (288.8–555.7)	
Neutered status	Intact	3	309.8 (301.4–849.1)	0.744
	Neutered	27	403.1 (341.7–574.0)	
Breed	Pure	24	384.9 (303.5–568.4)	0.432
	Mixed	6	480.5 (341.2–663.0)	
Body weight (BW)	BW < 15 kg	17	403.1 (320.5–592.9)	0.837
	BW ≥ 15 kg	13	384.9 (325.8–550.5)	
Immunophenotype	B-cell	25	384.9 (324.7–565.6)	0.448
	T-cell	5	527.0 (285.7–725.0)	
WHO clinical stage	II	1	186.4	0.042
	III	6	451.4 (300.0–739.8)	
	IV	20	384.9 (317.3–541.2)	
	V	3	849.1 (600.9–1196)	
Substage	a	18	403.5 (337.0–576.7)	0.787
	b	12	380.3 (311.0–582.4)	
Leukocytosis	No	21	403.1 (348.7–579.0)	0.372
	Yes	9	341.7 (227.8–579.0)	

**Table 4:** The serum YKL-40 levels of dogs with multicentric lymphoma in representative treatment time points.

Representative Time Points	Number of Cases	YKL-40 (pg/mL), Median (IQR)
Pretreatment	30	394.0 (332.2–576.7)
One week post-treatment	16	506.2 (370.7–660.9)
One week before progressive disease	7	353.4 (335.4–401.7)
Progressive disease	8	532.5 (340.6–661.4)
Post-treatment follow-up	6	256.8 (171.8–342.7)
Disease relapse	6	285.4 (256.0–453.8)

## Data Availability

The data presented in this study are available in this article.

## Supplementary Materials

The following supporting information can be downloaded at: https://www.mdpi.com/article/10.3390/ani14233391/s1, Table S1. The characteristics of 30 enrolled dogs with naïve multicentric lymphoma; Table S2. Treatment and outcome of 30 enrolled dogs with naïve multicentric lymphoma; Table S3. Serum YKL-40 value (pg/mL) at representative time points of 30 enrolled dogs with naïve multicentric lymphoma; Table S4. Blood examination at the time of pretreatment of 30 enrolled dogs with naïve multicentric lymphoma.
