# Supplementary material for: Evaluation of Serum YKL-40 in Canine Multicentric Lymphoma: Clinical and Diagnostic Implications
Source: Animals (Basel). 2024 Nov 25;14(23):3391. doi: 10.3390/ani14233391 (PMC11639910; doi:10.3390/ani14233391)
Supplement: Supplementary file 1 [file animals-14-03391-s001.zip › animals-3237368-supplementary.pdf]

Evaluation of Serum YKL-40 in Canine Multicentric Lymphoma: Clinical and Diagnostic Implications

Supplementary materials

Table S1. The characteristics of 30 enrolled dogs with naïve multicentric lymphoma.

Case	Breed	Sex (M=male, F=female)	Age (year)	BW (kg)	Clinical Stage	Substage	Pathological diagnosis	Flow cytometry with phenotype of medium to large lymphocyte
1	French bulldog	M	11	11.3	III	a	DLBCL	
2	Old English Sheepdog	M	12	31.2	III	b		T-cell
3	Chihuahua	M	13	4.2	IV	a		T-cell
4	Beagle	M	13	12.9	III	a		B-cell
5	English Bulldog	F	13	22.6	IV	a		B-cell
6	Golden retriever	M	9	37.1	IV	a		B-cell
7	Yorkshire Terrier	F	11	1.6	IV	a		B-cell
8	Miniature poodle	F	7	19.6	IV	a		B-cell
9	Chihuahua	F	9	3.1	II	a		T-cell
10	Mixed	F	10	7.4	IV	a		B-cell
11	Mixed	F	10	13.3	IV	b		B-cell
12	Welsh Corgi	M	8	15.6	IV	b		B-cell
13	Mixed	F	12	6.1	V	b		T-cell
14	Mixed	M	4	15.1	V	b	PTCL-NOS	

15	Mixed	F	13	15.3	IV	b		B-cell
16	Maltese	F	11	3.4	IV	a	DLBCL	
17	Welsh Corgi	M	10	14.6	III	a	DLBCL	
18	Mixed	M	13	19.1	IV	a	DLBCL	
19	Pomeranian	M	5	6.4	IV	a		B-cell
20	Miniature poodle	F	11	8	III	a		B-cell
21	Welsh Corgi	F	7	16.3	IV	a		B-cell
22	Welsh Corgi	M	3	14	IV	a		B-cell
23	ChowChow	M	11	19	IV	b		B-cell
24	Dachshund	F	15	4.5	IV	b		B-cell
25	Welsh Corgi	M	12	13.9	IV	b	DLBCL	
26	Welsh Corgi	M	8	15.5	IV	b		T-cell
27	Welsh Corgi	M	6	17.4	IV	a		B-cell
28	Welsh Corgi	M	9	14	IV	a		B-cell
29	Sheltie	M	11	13.8	V	b		B-cell
30	Husky	M	9	49.2	III	b		B-cell

Table S2. Treatment and outcome of 30 enrolled dogs with naïve multicentric lymphoma.

Case	Treatment regimen	Treatment duration (days)	Complete Treatment, (Y=yes, N=no)	Disease relapse during study period (Y=yes, N=no)	Outcomes during study period	Progression free survival (days)	Follow-up time	Overall survival
1	CHOP	424	N	N	cancer	424	424	424
2	CHOP	117	Y	Y	cancer	195	347	347
3	CHOP	133	Y	N	alive	910	910	910
4	LHOP	105	Y	N	Seizure	434	434	434
5	CHOP	143	N	N	cancer	143	143	143
6	CHOP	139	Y	Y	cancer	185	224	224
7	CHOP	189	Y	N	renal failure	1120	1120	1120
8	CHOP	141	Y	Y	cancer	320	351	351
9	CHOP	210	Y	N	alive	1398	1398	1398
10	CHOP	210	Y	N	pneumoia	533	533	533
11	CHOP	203	Y	Y	cancer	554	560	560
12	CHOP	196	Y	Y	alive	356	1142	1142
13	CHOP	49	N	N	cancer	49	49	49
14	CHOP	113	Y	Y	cancer	218	413	413
15	LHOP	93	N	N	cancer	93	93	93
16	CHOP	167	N	N	cancer	167	167	167
17	LHOP	178	Y	N	alive	925	925	925
18	CHOP	234	N	N	cancer	234	234	234
19	CHOP	112	Y	Y	cancer	155	155	155

20	LHOP	104	Y	Y	cancer	279	645	645
21	CHOP	126	Y	Y	alive	213	213	213
22	LHOP	115	Y	Y	cancer	269	534	534
23	No treatment							
24	CHOP	175	Y	Y	cancer	211	267	267
25	LHOP	98	Y	Y	cancer	237	241	241
26	LHOP	102	Y	Y	cancer	196	350	350
27	LHOP	91	Y	Y	alive	287	441	441
28	CHOP	164	Y	Y	cancer	252	309	309
29	CHOP	168	Y	Y	alive	209	209	209
30	No treatment							

Table S3. Serum YKL-40 value (pg/mL) at representative time points of 30 enrolled dogs with naïve multicentric lymphoma.

	Representative time points					
Case	Pre-treatment	Post one week of treatment	One week before progressive disease[1]	Progressive disease[1]	Post-treatment follow-up	Relapse
1	146.1		335.4	410.6		
2	527	339.3	264.2	679.9		
3	403.1	545			109.2	
4	351.3	503			312.3	
5	165.4					
6	574					318.9
7	551.5	650.8				
8	309.8					
9	186.4		667.5	551.2		
10	557.1	509			288.5	
11	339.6					
12	445.2	347.5			192.7	227.9
13	600.9	813.3	353.4	317.3		
14	849.1	1278	401.7	652.8	433.9	858.4
15	341.7	503.3	378.9	264.4		
16	346	664.2	346	664.2		
17	1102	440.1			225	
18	403.9	245		513.8		
19	584.9					

20	619	338.1	
21	384.9		
22	510.4	512.9	265.3
23	173.7		
24	281.8		
25	301.4	1161	
26	384.9	458.4	270
27	659.1		
28	366.2		300.8
29	1196		
30	375.7		

[1]Progressive disease (PD) was defined as the appearance of one or more new lesions or at least a 20% increase in the sum of the longest diameters of up to five affected lymph nodes, according to response evaluation criteria for peripheral nodal lymphoma in dogs (V1.0) of the Veterinary Comparative Oncology Group (VCOG).

Table S4. Blood examination at the time of pre-treatment of 30 enrolled dogs with naïve multicentric lymphoma.

Case	White blood cells count(/μL)	Neutrophils Count (/μL)	lymphcytes count	Monocyte count count (/μL)	PCV (%)
1	5700	4047	969	627	38
2	7100	4260	2627	71	46.9
3	6786	4343	2172	271	36.1
4	13600	10472	1360	1224	39.2
5	24300	20412	2187	1701	40.3
6	11700	8073	2925	585	34
7	10064	6944	3019	101	28.4
8	7800	5694	1638	468	36.7
9	7500	5400	1725	150	56
10	131860	21098	108125	2637	22.3
11	5600	4424	616	448	29.6
12	5826	1690	990	3146	36.3
13	47200	14160	31152	1416	32.2
14	14000	8680	3920	1400	26.4
15	23300	7689	14446	699	43.7
16	8387	6542	1090	671	23.4
17	7600	5600	1300	700	43.4
18	9200	6992	92	644	39.1
19	10121	7287	1113	1113	24.4
20	10900	8393	1962	327	46.7
21	19300	17563	579	1158	39.2

22	7100	3976	1704	568	38.9
23	27900	25947	1395	558	21
24	20400	17136	1836	816	36
25	28900	24565	2023	1156	41.9
26	7000	4690	1470	490	49.9
27	20600	8446	11948	206	32.6
28	15800	13272	1264	1264	39.2
29	7800	5700	1400	700	38
30	8400	6636	924	2520	33.8
